# Estimated distribution of malaria cases among children in sub-Saharan Africa by specified age categories using data from the Global Burden of Diseases 2019

**DOI:** 10.1186/s12936-023-04811-z

**Published:** 2023-12-05

**Authors:** Olorunfemi A. Oshagbemi, Pedro Lopez-Romero, Cornelis Winnips, Katalin R. Csermak, Guoqin Su, Elodie Aubrun

**Affiliations:** 1 Novartis Pharma AG, Basel, Switzerland; 2 Novartis Pharmaceuticals Corporation, East Hanover, NJ USA

**Keywords:** Child health, Malaria, *Plasmodium falciparum*, Epidemiology, Sub-saharan Africa, Disease burden, Diversity and inclusion

## Abstract

**Background:**

Children in sub-Saharan Africa (SSA) remain the most vulnerable to malaria and malaria mortality. This study estimated the disease burden and distribution of *Plasmodium falciparum* malaria among children with age categories (0 to < 2 years, 2 to < 6 years, 6 to < 12 years, ≥ 12 years) in SSA.

**Methods:**

Data on the number of cases and incidence rates of *P. falciparum* malaria by age group from the Institute of Health Metrics and Evaluation (GBD 2019) for 11 countries in SSA was employed in this study. The best-fitting distribution of *P. falciparum* malaria cases by prespecified age categories was derived using a combination of a Log-normal and Weibull distribution.

**Results:**

*Plasmodium falciparum* malaria was 15.4% for ages 0 to < 2 years, 30.5% for 2 to < 6 years, 17.6% for 6 to < 12 years, and 36.5% for ≥ 12 years based on data from countries in SSA. The results have important implications for the current drive by the FDA and EMA to ensure the representativeness of real-world populations in clinical trials evaluating the safety and efficacy of medication exposure.

**Conclusions:**

The theoretical distributions of *P. falciparum* malaria will help guide researchers in ensuring that children are appropriately represented in clinical trials and other interventions aiming to address the current burden of malaria in SSA.

## Background

Malaria remains a major public health challenge globally, especially in low and medium-income countries, putting close to half of the world’s population at risk of infection [1]. Children are predominantly susceptible to malaria infections, unlike adults that have grown up in endemic regions, as they have yet to acquire the needed immunity to defend themselves against the disease. The malaria parasite infects children, destroying red blood cells exponentially, and leading to fever, vomiting, diarrhoea, and anaemia [2]. If not treated within 24 h, malaria can progress to severe illness, including convulsions and coma, which can lead to death [3]. In 2021, there were over 247 million estimated cases of malaria in 85 malaria-endemic countries, with over 619,000 deaths worldwide, a 12% increase from 2019 [4]. The African region accounted for approximately 95% of cases and 96% of deaths globally, furthermore, 80% of all malaria deaths in this region are among children under 5 years [4]. Of all malaria parasite species known to infect humans, *Plasmodium falciparum* is the most prevalent form in Africa and is predominantly responsible for severe malaria among children [5].

Researchers have reported various estimates for malaria incidence and prevalence rates among children [6]. The differences in estimates reported have been attributed to several factors namely, study inclusion, malaria case definition, transmission intensity, and seasonality [2]. Importantly, accurate knowledge of the distribution of malaria in age groups is an important tool in planning, implementing, and evaluating malaria interventions [7]. A report from the first sub-Saharan African (SSA) summit meeting on malaria cites a ‘dire lack of extensive and comparable data about malaria’, and calls, amongst other things, for more research on trends in incidence and prevalence, epidemic outbreaks, and clinical epidemiology [7].

As outlined by the United States Food and Drug Administration (FDA), adequate representation of populations in clinical trials and studies supporting regulatory submissions helps ensure that the data generated in the development program reflect the diversity of the population expected to use the medical product (if approved) and as well as potentially differential impacts on effectiveness and safety outcomes in these population [8]. Despite policies, guidelines, and regulations to promote the diversification of clinical trials by the European Medicines Agency (EMA) and the FDA, the population included in clinical research continues to be less than proportionate to their representation in the real world [9]. Children are particularly vulnerable to malaria infections and death, thus understanding the distribution of malaria within this subpopulation is of prime importance to including them in clinical trials and other interventions aimed at reducing the burden of malaria. However, there is currently limited information on the distribution of malaria by age group among children. Consequently, this study aims to estimate the distribution of *P. falciparum* malaria among children by specified age categories (0 to < 2 years, 2 to < 6 years, 6 to < 12 years, ≥ 12 years) in SSA. These age categories were chosen as they are also the age bands commonly used to provide an age/body-weight specific dose of anti-malarial drugs such as artemether-lumefantrine (Coartem®).

## Methods

### Data source and malaria case definition

Secondary data from the Global Burden of Diseases 2019 (GBD 2019), conducted by the Institute of Health Metrics and Evaluation (IHME) was used in this study. IHME collects malaria information using various approaches and in collaboration with various stakeholders globally. The malaria information available from IHME/GBD includes the incidence, prevalence, and mortality rates. Information on the number of cases and incidence rates of *P. falciparum* malaria by age group as defined by IHME was used in this study (source data: IHME/GBD). IHME/GBD condition detail notes define malaria as an acute parasitic mosquito-borne disease. An individual experiences malaria if he/she presents 1 to 2 weeks of persistent fever, chills/shivering, sweating, joint pains, and headache. The individual will likely be lethargic and feverish, with a resultant loss of daily function during the attack. Individuals with an untreated *P. falciparum* malaria infection may develop severe malaria, which includes the symptoms of malaria plus potential swelling, difficulty breathing, unconsciousness, and death. The source data did not allow specify if the malaria was uncomplicated or severe malaria. More information on the data is available via the data information sheet from the IHME [10].

### Study population

The analyses focused on 11 countries in SSA including Mali, Burkina Faso, Ivory Coast, Niger, Uganda, Rwanda, Kenya, Gabon, Angola, the Democratic Republic of Congo (DRC), and Nigeria. These locations were selected *a priori* as potential sites for clinical trials. The selected countries adequately allowed us to estimate the distribution of *P. falciparum* malaria in the age groups of interest. The IHME provided information on the *P. falciparum* malaria cases, incidence rate, and population for defined age groups for all countries of interest (Table 1). Data from the 11 countries were pooled together to come up with a weighted average employed in the robust estimation of the distribution of malaria cases in the age categories of interest. In this overall population, the number of malaria subjects in each category of age was obtained as the weighted average of the number of malaria subjects in each observed age category, weighted by the total population in the respective category of age in each country.

**Table 1:** Proportion of malaria cases overall by age interval

Age category of interest^a^	Empirical (%)	Gamma (%)	Weibull (%)	Log-normal (%)	Exponential (%)
0 to < 1	0.060	0.076	0.079	0.055	0.081
1 to < 5	0.343	0.262	0.262	0.344	0.263
5 to < 10	0.172	0.229	0.227	0.227	0.226
10 to < 15	0.143	0.151	0.149	0.119	0.148
15 to < 20	0.090	0.099	0.098	0.070	0.097
20–95	0.192	0.184	0.185	0.170	0.185

^a^Age in years

### Statistical approach

Using the data obtained from IHME for different predefined age groups relative to the age groups desired for this review, four competing theoretical distributions (Gamma, Weibull, exponential, and Log-normal) were fitted to the overall empirical distribution of age in the malaria patients stratified by given categories of age defined in Table 1.

The Mean Squared Error (MSE) was used to select the best-fitting distribution among the 4 different competing ones. The MSE, calculated as the average squared difference between the observed proportions and the ones predicted by the distribution, provides a measure of the accuracy of the different options, as the deviation from the theoretical with respect to the empirical distribution (i.e., observed proportions).

The MSE was calculated as

$$100 \times \sqrt{\frac{\sum _{i=1}^{n}{\left({O}_{i}-{E}_{i}\right)}^{2}}{n}}$$

where $i$ indicates the order of selected category of age ($n=6$) as shown in Table 1, ${O}_{i}$ is the number of individuals observed for category $i$ and ${E}_{i}$ is the number of individuals estimated for a given theoretical distribution. A theoretical distribution with the lower MSE will be the one with the smallest deviation with respect to the observed empirical data. The best-fitting theoretical distribution was used to estimate the percentage of the population for the different age intervals of interest (0 to < 2, 2 to < 6, 6 to < 12, ≥12 years). Parameters of the theoretical distributions were estimated by Maximum Likelihood (ML), using the *MASS package* of *R version 4.0.2*.

## Results

**Fig. 1:**
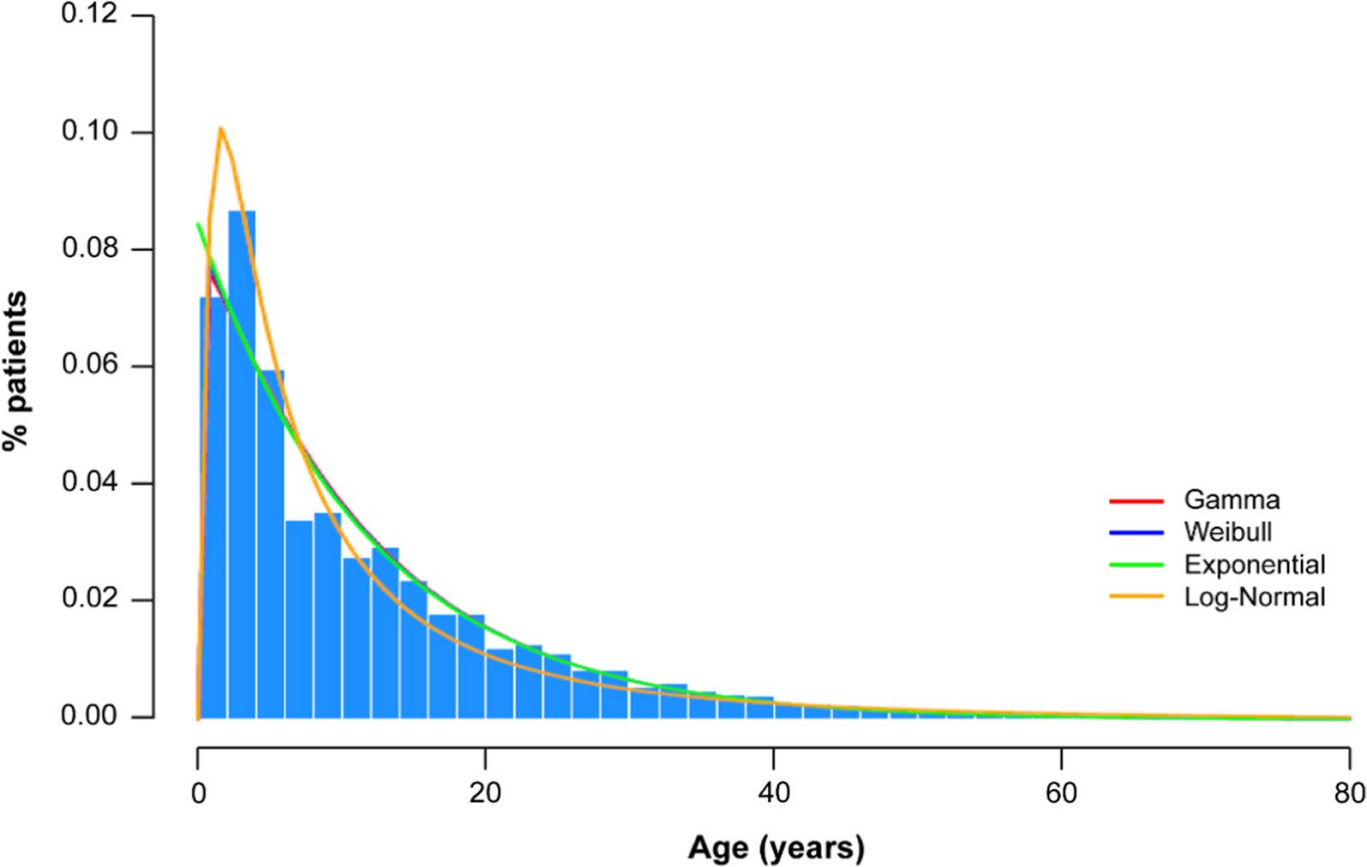
The proportions observed for the different categories of age, as well as the proportions predicted for the different models. The ML estimates for the parameters of the 4 competing distributions were Gamma: Shape = 1.0305 and rate = 0.0869, Weibull: Shape = 1.007 and scale = 11.8898, Exponential: rate = 0.0844, Log-normal: mean = 1.9144 and standard deviation = 1.1995. *The line of the models overlap in some regions and the difference are difficult to see. The bar graphs are the empirical (actual) data, corresponding to the overall distribution of all age groups described above

In general, the observed individual distributions of *P. falciparum* malaria in the 11 countries using GBD 2019 data varied by country, and the distributions were fitted to a weighted overall distribution. The percentages estimated from the Gamma, Weibull, Log-normal, and exponential distributions provided slightly different proportions of *P. falciparum* malaria for the different categories of the given ages (see Table 1; Fig. 1). The ML estimates for the parameters of the 4 competing distributions were Gamma: Shape = 1.0305 and Rate = 0.0869, Weibull: Shape = 1.007 and Scale = 11.8898, Exponential: Rate = 0.0844, Log-normal: Mean = 1.9144 and Standard deviation = 1.1995. Figure 1 shows the histogram of the empirical data, corresponding to the overall weighted distribution of the 11 countries, superimposed with the density function of the 4 fitted distributions. The density functions of the model overlap in some regions of the curve, making it difficult to see each distribution distinctively. Table 1 shows the estimated proportions by age category for the Gamma, Weibull, Log-normal, and exponential distributions. As noticed, both in results shown in Table 1 and in Fig. 1, that the Log-normal distribution predicts the empirical (observed) proportions of individuals at the lower tail of the distribution (e.g., [0 to < 1[ and [1 to < 5] years), much better than the other competing distributions. On the contrary, either the Gamma or the Weibull distributions perform much better than the Log-normal in the intervals for ages ≥ 10 years. In the interval of 5–10 years, all the theoretical distributions slightly overestimate the observed empirical proportion. Based on these observations, combinations of a Gamma with Log-normal and Weibull with Log-normal were also considered. For the combination of these distributions, the Log-normal was used to predict the categories of age 0 to < 1 and 1 to < 5 years, while the Gamma (or Weibull) was used to predict the categories of age 10 to < 15, 15 to < 20 and 20 to < 95 years. Since the probability density function needs to integrate to 1, the category of age (5 to < 10 years) was calculated as the difference to 1 with respect to the other five categories of age shown in Table 1.

Furthermore, estimation of the proportion of *P. falciparum* malaria cases by age categories of interest according to the theoretical distributions was conducted. The estimated proportions of *P. falciparum* malaria cases were compared to the observed ones to calculate the MSE. The MSE are shown in Table 2. According to the MSE, the combination of Log-normal/Weibull distributions, as discussed above, is the best fitting distribution. The proportions estimated from the Gamma, Weibull, Log-normal, Log-normal/Gamma combination, and Log-normal/Weibull combination for the 4 categories of the age of interest are shown in Table 3. The categories of age of interest are 0–1.9, 2–5.9, 6–11.9, and ≥ 12 years, respectively. To calculate the proportion of malaria cases for these 4 categories of age using the combination of the Log-normal and Gamma distributions, the Log-normal distribution was used for the age intervals 0–1.9 and 2–5.9 years, and the Gamma distribution for the interval ≥ 12 years. Since the proportion of the total population needs to add up to 1, the category 6–11.9 years was obtained as the difference of the total of the other three categories from 1. The same was done for the combination of the Log-normal and Weibull distributions. Because of rounding, the total for the Gamma, Weibull, and Log-normal might not add to 1. Please, note that the best- fitting distribution can be used to estimate the percentage of *P. falciparum* malaria cases for any other intervals of the age of interest.

**Table 2:** Mean squared error (overall population)

Distribution	MSE
Gamma	4.138
Weibull	4.102
Log-normal	2.742
Exponential	4.058
Log-normal/Gamma combination	0.658
Log-normal/Weibull combination	0.554

*MSE* mean squared error

**Table 3:** Predicted proportions of malaria cases in the overall population by age category of interest

Age category of interest^a^	Gamma (%)	Weibull (%)	Log-normal (%)	Log-normal/Gamma (%)	Log-normal/Weibull (%)
0–1.9	14.91	15.30	15.43	15.43	15.43
2–5.9	24.25	24.18	30.50	30.50	30.50
6–11.9	24.32	24.08	22.35	17.56	17.63
≥ 12	36.52	36.45	31.72	36.52	36.45

^a^Age in years

## Discussion

This study found that the best-fitting estimate of the distribution of *P. falciparum* malaria cases by prespecified age categories was derived using a combination of a Log-normal and Weibull distribution. Consequently, the distribution of *P. falciparum* malaria was 15.4% for ages 0 to < 2 years, 30.5% for 2 to < 6 years, 17.6% for 6 to < 12 years, and 36.5% for ≥ 12 years based on data from countries in SSA.

Researchers have reported a wide range of estimates on the distribution of malaria in children in Africa. Furthermore, most of these studies have aggregated children into wide age brackets, making extrapolation of the distribution of malaria by specific age groups challenging. For instance, a survey conducted among households with 553 children (6–59 months) in Benin, reported a prevalence of up to 68% which slightly varied between the three survey visits [11]. Furthermore, a four quarterly survey of 7,000 children less than 10 years old in Ethiopia found that the incidence rate varied between 2.4 and 20% in high-altitude areas, depending on the quarter of the year [12]. A study conducted in Mali among 1401 participants (over 65% less than 20 years) from 2012 to 2020, reported significantly different estimates at the start and end of the rainy season, demonstrating the impact of seasonality/transmission intensity on malaria distribution [13]. The disparity in published estimates can be attributed to differences in patient inclusion, malaria case definition, assumptions made in the estimation, differences in seasonality, and transmission intensity [2]. Most studies reporting the incidence of malaria among children are cross-sectional by design, making them less robust in evaluating the incidence of the disease condition; also, surveys are generally more prone to non-response and recall biases [14]. In addition, these studies are usually conducted in a single centre, which raises questions about their generalizability in other centres not to mention an entire country/region.

Until recently, malaria was thought of as a rural area disease due to the fact that transmitting vectors were said to propagate more in rural communities [15]. A systematic review estimating the burden of malaria among children under 5 years of age observed that when the urban–rural ratios were modified, total malarial fever estimates remained similar [2], suggesting that significant changes in this assumption do not have a major effect on estimates reported in this study. Limited resources, adequate infrastructural amenities, and rates of urbanization have resulted in poor housing, sanitation, and drainage systems, which could increase vector survival (particularly *Anopheles stephensi*) and subsequent human contact [15]. In addition, valid operational definitions of incidence rates and prevalence proportions are important foundational components for understanding and monitoring diseases. Most researchers evaluating the burden of malaria among children do not clearly define key parameters under evaluation [16].

Limited data on the distribution of *P. falciparum* malaria by specific age categories has constrained efforts aimed at evaluating the safety, efficacy, and dosing of potential treatments for specific age groups needed to address the burden of malaria among children. Previous studies that have tried to establish the distribution of malaria among children in SSA have aggregated children into broad age categories, making targeted interventions among children of specific age groups challenging [17]. While others have focused on congenital and/or neonatal malaria [18–20]. This study is the first of its type to use secondary data from the IHME (GBD 2019) and a unique approach to estimate the distribution of malaria among precisely defined age groups. There are currently ongoing efforts via the recently established continental database of malaria surveys that aims to allow researchers to elucidate empirical distribution for malaria distribution at a regional level for malaria control and health service provision for countries in SSA [7]. In the long term, these initiatives will also need to focus on establishing the distribution of malaria in SSA by age, which will in turn help establish equitable health service provisions and targeted interventions for vulnerable groups.

## Study strengths and limitations

Firstly, this study used data from 11 countries in SSA, which might have limited the robustness of the estimated distribution of malaria cases by age. This study pools real-world data from different countries to ascertain the malaria distribution among children of specified age groups. Subsequent studies should consider including data from a greater number of countries within SSA. The most recent information available on the distribution of malaria from the IHME (GBD 2019) by the time the authors conceived was used in this study, which is known to be a reliable source of malaria information globally. It is also important to note that IHME generates information from various sources, such as the Malaria Atlas Project, surveys of households, interviews, and publications. Due to the paucity of useful data available for estimating the burden of malaria, IHME (GBD 2019) remains a valuable source. Furthermore, a plausible and pragmatic approach in estimating the distribution of malaria in this study. This unique approach to estimate the distribution of disease by age groups can be applied to similar data. In this study, it could not be ascertained if the cases of malaria were uncomplicated or severe cases in the evaluated population. Notwithstanding the caveats, this study is the first to report the distribution of malaria among children using data from 11 African countries. The findings from this study have the potential to help mitigate the current challenges encountered in clinical trials which include, setting appropriate enrollment targets (for the number of children in malaria clinical trials, which are representative of children in the general population) and lack of evidence-based drug dosing or off-label use of drugs in children [21–23], and will potentially encourage publication of age-specific safety and efficacy findings on important outcomes among children [24]. Lastly, the result from this study is based on the assumption that the 11 countries included offer a representative mix of different transmission intensities and seasonal variabilities of malaria.

## Conclusions

Using secondary data from IHME (GBD 2019), the distribution of *P. falciparum* malaria cases among children by prespecified age groups was estimated. The results have important implications for the current drive by the FDA and EMA to ensure the representativeness of real-world populations in clinical trials evaluating the safety and efficacy of medication exposure. The theoretical distributions of *P. falciparum* malaria reported in this study will help guide researchers in ensuring that children are appropriately represented in clinical trials and other interventions aiming to address the current burden of malaria in SSA.

## Data Availability

Data can be made available upon reasonable request from researchers whose proposed use of the data has been approved OR Research data will not be shared due to privacy restrictions.

## Abbreviations

EMA: European Medicines Agency
FDA: Food and Drug Administration
GBD: Global Burden of Diseases
IHME: Institute of Health Metrics and Evaluation
ML: Maximum likelihood
MSE: Mean squared error
*P. falciparum*: *Plasmodium falciparum*
SSA: sub-Saharan Africa
